# Pathogenic Mitochondrial tRNA Point Mutations: Nine Novel Mutations Affirm Their Importance as a Cause of Mitochondrial Disease

**DOI:** 10.1002/humu.22358

**Published:** 2013-08-14

**Authors:** Emma L Blakely, John W Yarham, Charlotte L Alston, Kate Craig, Joanna Poulton, Charlotte Brierley, Soo-Mi Park, Andrew Dean, John H Xuereb, Kirstie N Anderson, Alistair Compston, Chris Allen, Saba Sharif, Peter Enevoldson, Martin Wilson, Simon R Hammans, Douglass M Turnbull, Robert McFarland, Robert W Taylor

**Affiliations:** 1 Wellcome Trust Centre for Mitochondrial Research, Institute for Ageing and Health, Newcastle UniversityNewcastle upon Tyne, UK; 2 Nuffield Department of Obstetrics and Gynaecology, University of OxfordOxford, UK; 3 Department of Neurology, The West Suffolk Hospital, Bury St EdmundsSuffolk, UK; 4 Department of Clinical Genetics, East Anglian Medical Genetics Service, Addenbrooke’s HospitalCambridge, UK; 5 Department of Pathology, University of CambridgeCambridge, UK; 6 Department of Neurology, Royal Victoria InfirmaryNewcastle upon Tyne, UK; 7 Department of Clinical Neurosciences, University of CambridgeCambridge, UK; 8 Department of Neurology, Addenbrooke’s HospitalCambridge, UK; 9 West Midlands Regional Clinical Genetics Unit, Birmingham Women’s NHS Foundation TrustBirmingham, UK; 10 The Walton Centre NHS Foundation TrustLiverpool, UK; 11 Wessex Neurological Centre, University Hospitals SouthamptonSouthampton, UK

**Keywords:** mitochondrial tRNA, single-fiber studies, mitochondrial disease, segregation

## Abstract

Mutations in the mitochondrial genome, and in particular the mt-tRNAs, are an important cause of human disease. Accurate classification of the pathogenicity of novel variants is vital to allow accurate genetic counseling for patients and their families. The use of weighted criteria based on functional studies—outlined in a validated pathogenicity scoring system—is therefore invaluable in determining whether novel or rare mt-tRNA variants are pathogenic. Here, we describe the identification of nine novel mt-tRNA variants in nine families, in which the probands presented with a diverse range of clinical phenotypes including mitochondrial encephalomyopathy, lactic acidosis, and stroke-like episodes, isolated progressive external ophthalmoplegia, epilepsy, deafness and diabetes. Each of the variants identified (m.4289T>C, *MT-TI*; m.5541C>T, *MT-TW*; m.5690A>G, *MT-TN;* m.7451A>T, *MT-TS1;* m.7554G>A, *MT-TD;* m.8304G>A, *MT-TK;* m.12206C>T, *MT-TH;* m.12317T>C, *MT-TL2;* m.16023G>A, *MT-TP*) was present in a different tRNA, with evidence in support of pathogenicity, and where possible, details of mutation transmission documented. Through the application of the pathogenicity scoring system, we have classified six of these variants as “definitely pathogenic” mutations (m.5541C>T, m.5690A>G, m.7451A>T, m.12206C>T, m.12317T>C, and m.16023G>A), whereas the remaining three currently lack sufficient evidence and are therefore classed as ‘possibly pathogenic’ (m.4289T>C, m.7554G>A, and m.8304G>A).

## Introduction

Mutations in the mitochondrial genome (mtDNA) give rise to a clinically and biochemically heterogeneous range of genetic disorders affecting children and, perhaps more significantly, adults [Schon et al., 2012; Tuppen et al., 2010]. Both the diagnosis of mitochondrial disease and the molecular characterization of underlying pathogenic mutations are complicated by the variability in penetrance and tissue specificity observed even between close family members, as well as the lack of a consistent genotype–phenotype correlation [McFarland et al., 2010; Ylikallio and Suomalainen, 2012]. Comprehensive databases of reported mutations, polymorphisms and their supporting data (e.g., MitoMAP, www.mitomap.org [Ruiz-Pesini et al., 2007]) as well as tools such as pathogenicity scoring systems [Mitchell et al., 2006; Yarham et al., 2011] are therefore invaluable when determining the pathogenic role of mtDNA sequence variants [Elson et al., 2012].

Determining the pathogenic nature of a novel or rare mtDNA variant is particularly problematic for mt-tRNA variants. Mt-tRNA point mutations have been associated with a diverse range of clinical phenotypes including epilepsy, deafness, diabetes, ophthalmoparesis, myopathy, cardiomyopathy, and encephalopathy [Florentz et al., 2003; Yarham et al., 2010]. Over 200 mt-tRNA point mutations have been linked to mitochondrial disease but less than half of these have sufficient evidence for a classification of “definitely pathogenic” [Yarham et al., 2011]. Although some mt-tRNA mutations are found relatively often in patients (e.g. m.3243A>G and m.8344A>G), most are rare, having only ever been identified in one or two families [Ruiz-Pesini et al., 2007; Yarham et al., 2010]. Mt-tRNA point mutations typically cause a loss of mt-tRNA stability leading to defective mitochondrial translation and a combined respiratory chain deficiency. Potential mechanisms include aberrant processing of the mRNA transcript by RNases P and Z^L^, impaired post-transcriptional mt-tRNA modification (e.g., specific base modifications, 3′-end addition of the –CCA sequence and mt-tRNA aminoacylation) and compromised interaction of the mt-tRNA with both mtEF-Tu (mitochondrial elongation factor Tu) and the mitoribosome [Levinger et al., 2004].

Determining whether an identified mt-tRNA variant is in fact the pathogenic cause of the observed clinical phenotype is difficult, partly as a result of the observed genotype–phenotype heterogeneity in mitochondrial disease. However, the reliability of such assessment has been improved by the development and revision of a pathogenicity scoring system consisting of a number of weighted criteria [Yarham et al., 2011]. The gold-standard studies in particular provide crucial functional evidence; for heteroplasmic mutations, single-fiber studies investigate mutation segregation with biochemical deficiency, and for homoplasmic mutations, Northern blot analyses assess variations in mt-tRNA steady-state levels [McFarland et al., 2004].

It was speculated over a decade ago that we might soon be “scraping the bottom of the barrel” with respect to identifying novel pathogenic mtDNA mutations and elucidating their importance in human pathology [DiMauro and Andreu, 2000]. However, in this study, we present clinical, histochemical, and molecular genetic data confirming the identification of novel heteroplasmic mt-tRNA variants in each of nine unrelated patients who present with a diverse range of clinical features. Based on the robust criteria of the pathogenicity scoring system, the evidence we present here confirms six of these variants as “definitely pathogenic” and the remaining three as “possibly pathogenic,” affirming mt-tRNA mutations as an important cause of human mitochondrial disease.

## Materials and Methods

### Patients

The patients presented here were identified in the clinic as having suspected mitochondrial disease and were referred to the UK NHS Highly Specialised Services Diagnostic Laboratory in Newcastle upon Tyne. Informed ethical consent was obtained for further testing through the acquisition of a skeletal muscle biopsy.

The clinical phenotypes for each of the nine patients studied are described in Table 1, with further details provided in the Supporting Information (Supp. Document S1). Each patient harbored a single, novel mt-tRNA variant in a different mt-tRNA gene, and there was considerable diversity in the clinical presentations of the disease. Family histories are indicated, along with the details of the mtDNA haplogroup of each patient and heteroplasmic mutation loads in a variety of patient and familial tissue samples (e.g., muscle, blood, urine, and buccal epithelial cells).

### Histology and Histochemistry

Standard histological (Haematoxylin & Eosin, modified Gomori trichrome staining) and histochemical (cytochrome *c* oxidase [COX], succinate dehydrogenase [SDH], and sequential COX–SDH) analyses of proband muscle biopsies (where available) were performed on fresh-frozen skeletal muscle sections (10 μm), according to established protocols [Old and Johnson, 1989]. Individual COX-positive and COX-deficient fibers were isolated by laser microdissection and lysed to obtain total cellular DNA for single-fiber mutation segregation studies.

### Molecular Genetic Studies

Total DNA was extracted from all available tissues by standard procedures, and mtDNA rearrangements were excluded by long-range PCR. Direct sequencing of the entire mitochondrial genome was then performed on homogenate skeletal muscle DNA. Total DNA was additionally extracted from available tissues obtained with consent, from appropriate maternal relatives.

Known polymorphisms were excluded through the searching of the MitoMAP (www.mitomap.org) [Ruiz-Pesini et al., 2007] and mtDB (http://www.mtdb.igp.uu.se/) databases [Ingman and Gyllensten, 2006]. Haplogroup analysis was performed using Haplogrep (http://haplogrep.uibk.ac.at/) and Phylotree (http://www.phylotree.org/) [Kloss-Brandstätter et al., 2011; van Oven and Kayser, 2009].

### Assessment of Mutation load by Quantitative Pyrosequencing

The mtDNA mutation load in all available homogenate tissue DNA and individual COX-positive and COX-deficient fibers was assessed by quantitative pyrosequencing. The Pyromark Assay Design Software v.2.0 (Qiagen, Crawley, West Sussex, UK) was used to design locus-specific PCR and pyrosequencing primers (Supp. Table S1) for each variant and pyrosequencing was performed on the Pyromark Q24 platform according to the manufacturer’s protocol. Quantification of the heteroplasmy level of each variant was achieved using Pyromark Q24 software to directly compare the relevant peak heights of both the wild-type and mutant nucleotides at the relevant position [White et al., 2005].

### Quantification of mt-tRNA Steady-State Levels by High-Resolution Northern Blot

High-resolution Northern blotting to assess mt-tRNA^His^ steady-state levels in patient skeletal muscle was performed as previously described [Taylor et al., 2003]. Probes were generated by PCR amplification across the mt-tRNA^Leu(UUR)^ (75 bp) and mt-tRNA^His^ genes (69 bp). A 154 bp tRNA^Leu(UUR)^ probe was generated using the forward primer L3200 (positions 3,200–3,219) and the reverse primer H3353 (positions 3,353–3,334); a 120 bp mt-tRNA^His^ probe was generated using the forward primer L12086 (positions 12,086–12,104) and the reverse primer 12,205 (positions 12,205–12,186) (GenBank Accession number NC_012920.1 for human mtDNA).

**Table 1:** Genotypic and Phenotypic Data from all Nine Case Studies

						Mutation Load (%)			
Patient Details^a^ (Sex/Age of presentation)	Clinical Presentation^b^	Family History^c^	Mutation^d^	Haplogroup^e^	Muscle Biopsy findings^f^	Patient Tissues^g^	Familial Tissues^h^	Pathogenicity Classification^i^	Inheritance Pattern^j^
1 (F/11)	Retinopathy, diabetes, dysphagia; MRI shows cerebral atrophy	No	m.4289T>C (*MTTI*)	H1c1	“numerous” COX-deficient fibres and RRF	M: 39% B: 7% U: 7% BM: 10%	Unaffected mother and sisters—B: 0%; U: 0%; BM: 0%	7 points—Possibly Pathogenic	Sporadic
2 (M/30)	MELAS; stroke-like episodes and cortical blindness; MRI shows occipital lobe infarct	No	m.5541C>T (*MTTW*)	J1c3	75% COX-deficient fibres	M:84% U: 87%	Unaffected mother—U: 51%	11 points—Definitely Pathogenic	Maternal
3 (F/13)	CPEO, ptosis, proximal myopathy	No	m.5690A>G (*MTTN*)	H7	13% COX-deficient fibres, 5% RRF	M: 35% B: 0% U: 0%	Not tested	11 points—Definitely Pathogenic	N.D.
4 (M/15)	CPEO, ptosis	No	m.7451A>T (*MTTS1*)	H1	35% COX-deficient fibres, 19% RRF	M: 37% U: 3% BM: 0% B: 0%	Unaffected mother and sisters—B: 0%; U: 0%; BM: 0%	11 points—Definitely Pathogenic	Sporadic
5 (M/7)	Myopathy, ataxia, nystagmus, migraines, lactic acidosis	Mum and older sister unaffected; other sister has diagnosis of MS	m.7554G>A (*MTTD*)	H	40% COX-deficient fibres, 25% RRF	M: 87% B: 6% U: 22% BM: 10%	Unaffected mother—B: 0%; U: 0%; BM: 0%	9 points—Possibly Pathogenic	Sporadic
6 (M/7)	Epilepsy, ataxia, visual disturbance, deafness	No	m.8304G>A (*MTTK*)	K1a1a1	25% COX-deficient fibres, some RRF	M: 82% B: 18 U: 58%	Unaffected mother—B: 5%; U: 10%; unaffected sister—B: 0%; U: 0%	8 points—Possibly Pathogenic	Maternal
7 (M/21)	MELAS-like encephalopathy; bilateral optic atrophy	Yes – clinically-affected brother with identical presentation	m.12206C>T (*MTTH*)	H1	50% COX-deficient fibres, 1% RRF	M: 95% B: 1% U: 87% F: 0%	Unaffected mother—B: 0%; U: 12%; clinically-affected brother—B: 0%; U: 90%; unaffected sister—B: 1%; BM: 1%; U: 1%	16 points—Definitely Pathogenic	Maternal
8 (M/48)	CPEO, ptosis, myopathy, exercise intolerance, diabetes	No	m.12317T>C (*MTTL2*)	H13a2	40% COX-deficient fibres, 5% RRF	M: 89% B: 5%	Not tested	11 points—Definitely Pathogenic	N.D.
9 (F/35)	Migraine, pigmentary retinopathy, deafness, leukariosis on MRI	No	m.16023G>A (*MTTP*)	V4	65% COX-deficient fibres, 5% RRF	M: 86 B: 9% U: 36%	Unaffected mother—B: 1%; U: 7%	11 points—Definitely Pathogenic	Maternal

a Patient details include ID number, sex, and age at first presentation.

b A variety of clinical presentations were seen across the patients, and abbreviations include: CPEO (Chronic Progressive External Ophthalmoplegia), MELAS (Mitochondrial Encephalomyopathy, Lactic Acidosis and Stroke-like episodes) and MRI (magnetic resonance imaging).

c Where known, evidence of pathology in family members is reported.

d The 9 different mutations are all located in different mt-tRNAs.

e The haplogroup of each patient was determined using the freely available Haplogrep Software.

f The percentage of COX-deficient fibres and ragged-red fibres (RRFs) in the muscle biopsy were calculated.

g Mutation loads were determined in a variety of tissues from the patient. Tissues examined included skeletal muscle (M), blood (B), urine (U), buccal mucosa (BM) and fibroblasts (F).

h Mutation loads were determined in a variety of tissues from maternally-related individuals. Tissues examined included skeletal muscle (M), blood (B), urine (U), buccal mucosa (BM) and fibroblasts (F).

i Each mutation was assigned a pathogenicity score and correspondingly classified according to the revised scoring system (Yarham *et al*. 2011).

j The pattern of inheritance within the family for each mutation was determined where possible based upon mutation loads in familial tissues.

### Assigning Pathogenicity to mt-tRNA Variants

The pathogenicity classification of each of the mt-tRNA variants identified and characterized in this study, was assigned using an updated version of a previously validated scoring system [Yarham et al., 2011]. This pathogenicity scoring system employs a number of weighted criteria covering a range of molecular, functional, and genetic data, from which an overall pathogenicity score (out of a total of 20 points) can be obtained.

Variants were classified as “definitely pathogenic” with a score of ≥11 points including evidence from one or more of the “gold-standard” investigations: segregation of the variant with a biochemical deficiency in single-fibers, high-resolution Northern blotting analysis of mt-tRNA steady-state levels, or *trans*mitochondrial cybrid studies. Variants were classified as “probably pathogenic” with a score of ≥11 points but lacking evidence from a “gold-standard” investigation, “possibly pathogenic” with a score of 7–10 points and a “neutral polymorphism” with a score of ≤6 points.

## Results

### Histochemistry

**Figure 1:**
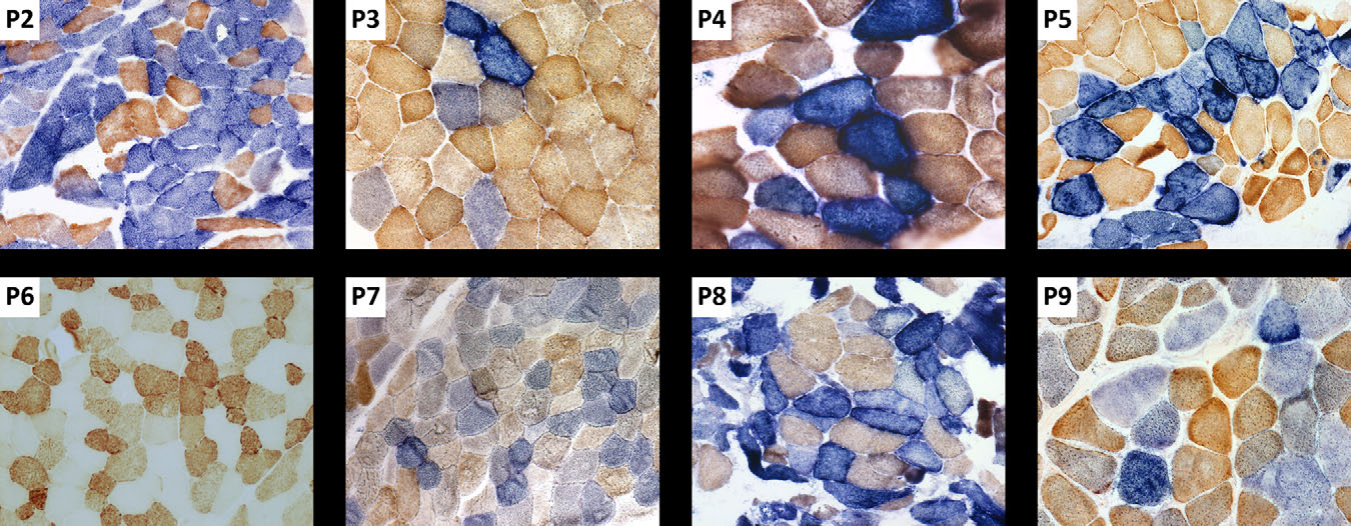
Sequential COX–SDH histochemistry. Sequential cytochrome *c* oxidase (COX) and succinate dehydrogenase (SDH) histochemistry was performed on skeletal muscle biopsies from all patients with the exception of patients 1 and 6. A mosaic pattern of COX activity is visible in each of the images, with COX-deficient fibers shown in blue and COX-positive fibers shown in brown. For patient 6, the individual COX histochemical reaction demonstrates a large number of COX-deficient fibers.

Dual enzyme histochemistry performed on each of the proband’s skeletal muscle biopsies where available, revealed a significant number of COX-deficient fibers (Fig. 1), several of which were accompanied by evidence of subsarcolemmal mitochondrial accumulation associated with ragged-red fibers, which is indicative of mitochondrial disease. For patient 6, muscle was not available to study using COX–SDH histochemistry but the individual COX reaction demonstrated a large number of COX-deficient fibers.

### Molecular Genetic Studies

Long-range PCR of skeletal muscle DNA samples from each of the nine patients excluded the presence of large-scale mtDNA rearrangements. Direct sequencing of the entire mitochondrial genome revealed a previously unreported substitution in an *MT-T* gene in each patient (Table 1), which appeared heteroplasmic on the chromatogram (Supp. Fig. S1). The substitutions were confirmed to be novel variants absent from the specific haplogroup of each proband (Table 1), and were not identified on databases of known polymorphisms.

**Figure 2:**
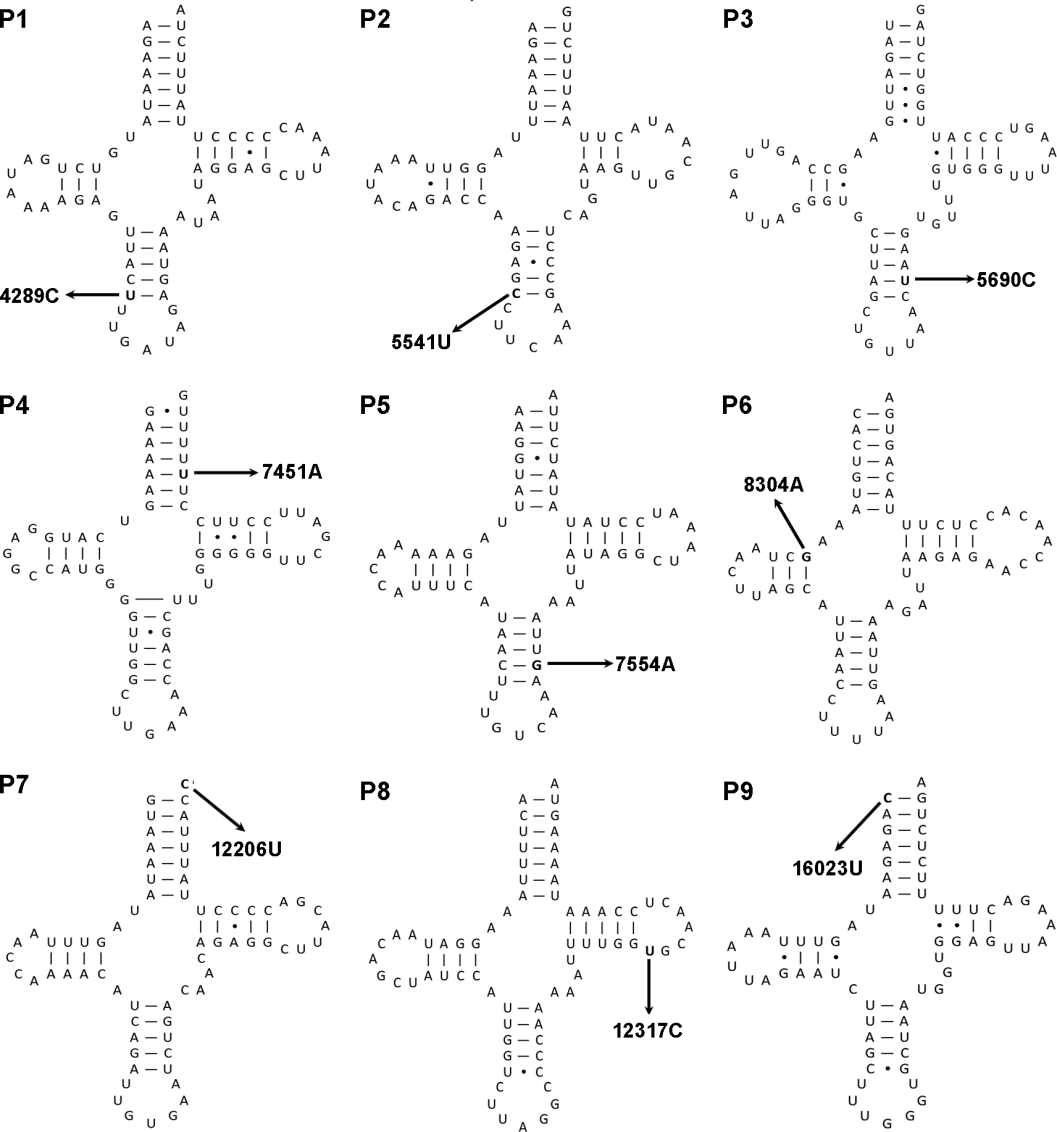
Location of mt-tRNA variants. The location of each of the identified mt-tRNA variants within the tRNA cloverleaf structure is shown. Each of the nine novel variants occurs within a different mt-tRNA, in a variety of stems, loops, and other regions. The affected position and the substitution that occurs are highlighted in bold.

The nine identified variants were each located within a different mt-tRNA and, as illustrated in Figure 2, are located in a variety of structural stem and loop regions. Each of the variants was found at a position that is highly conserved across evolution according to the consensus panel of species [Yarham et al., 2012], except for the m.4289T>C variant which shows moderate conservation and the m.7554G>A variant which shows poor conservation (Supp. Fig. S2). All nine novel mtDNA variants have been submitted for inclusion within the MitoMAP database.

### Mutation Heteroplasmy and Inheritance Patterns

Each of the nine identified variants was shown to be heteroplasmic through quantitative pyrosequencing analysis of mutation load in homogenate DNA from all available patient tissues. Each variant was assessed in skeletal muscle from the patient, whereas blood, urine, fibroblasts, and buccal mucosa were also tested where possible (Table 1).

The inheritance pattern of each variant was determined through an analysis of mutation load in noninvasive tissues (blood, urine, and buccal mucosa) from maternal relatives, where available. As described in Table 1, four of the variants were shown to be maternally inherited, three appear to be de novo and two remain undetermined due to a lack of familial samples for analysis.

### Segregation of mt-tRNA Mutations with Biochemical Deficiency

**Figure 3:**
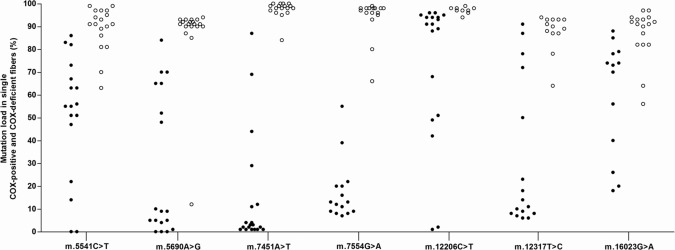
Single fiber mutation load segregation. This graph shows the mutation load measured in individual COX-positive (closed circles) and COX-deficient fibers (open circles) laser-micro dissected from a skeletal muscle biopsy taken from all the patients with the exception of patients 1 and 6, which were omitted due to a lack of available tissue. In each patient studied, the identified variant segregates with the biochemical deficiency.

Single-fiber analysis was performed to determine whether the mutation load in individual COX-positive and COX-deficient fibers correlated with the observed biochemical phenotype (Fig. 3) in each of the probands (with the exception of patients 1 and 6, from whom a muscle biopsy block was unavailable). Mutation loads were found to be significantly higher (*P* < 0.05, two-tailed *t* test) in the COX-deficient fibers (*n* ≥ 9) compared with the COX-positive fibers (*n* ≥ 10) (±SD) (Table 2), confirming segregation of the identified mtDNA variants with respiratory chain deficiency in each patient.

**Table 2:** Single Fiber Mutation Load Data

		Percentage mutation load				
Patient	mt-tRNA mutation	COX-positive fibers	COX-deficient fibers	*P* value
2	m.5541C>T	51.4 ± 28.1	90.0 ± 2.7	0.0003
3	m.5690A>G	27.9 ± 31.3	86.8 ± 18.3	<0.0001
4	m.7451A>T	14.6 ± 25.1	97.4 ± 3.8	<0.0001
5	m.7554G>A	17.5 ± 13.2	94.0 ± 8.7	<0.0001
7	m.12206C>T	73.8 ± 31.8	97.1 ± 1.5	0.0391
8	m.12317T>C	31.1 ± 32.4	87.7 ± 8.3	<0.0001
9	m.16023G>A	60.1 ± 25.3	86.9 ± 11.3	0.0005

The percentage mutation load in individual COX-positive and COX-deficient fibers from the seven patients investigated (plus or minus the standard deviation) is shown. All novel mt-tRNA variants showed significant segregation with the biochemical defect (*P* values) at the 95% confidence level.

### Quantification of mt-tRNA^His^ Steady-State Levels

Although statistically significant, the segregation of the m.12206C>T variant with biochemical deficiency in single fibers (Fig. 3) was less apparent than the segregation observed with the other variants. Consequently, high-resolution northern analysis of mt-tRNA^His^ steady-state levels was performed in addition to the single fiber analysis. The steady-state level of mt-tRNA^His^ was found to be ∼25% of the control mt-tRNA^His^ steady-state levels in this patient’s skeletal muscle (Supp. Fig. S3).

## Discussion

Mitochondrial tRNA point mutations are associated with a diverse range of clinical phenotypes that show only a limited correlation with genotype, and this considerable heterogeneity greatly complicates the diagnosis of mitochondrial disease. Here we describe the characterization of nine novel heteroplasmic mt-tRNA variants identified in nine unrelated patients. Each variant occurred in a different mt-tRNA and there was a range of associated clinical presentations including (but not limited to) epilepsy, diabetes, CPEO, and deafness (Table 1 and Supp. Document S1). This diversity reaffirms the heterogeneity seen in the phenotype–genotype relationship of mt-tRNA mutations, which arises due to the complex interactions between nuclear and mitochondrial genetics, the dramatic variations in tissue segregation of heteroplasmic mt-tRNA point mutations, and the threshold effect for pathogenicity. The importance of appropriate tissue selection is also demonstrated; for example, although the m.7554G>A variant in patient 5 was found at a level of 87% in skeletal muscle, it was detected at considerably lower levels in the other tissues investigated, with levels of 22% in urine, 10% in buccal mucosa, and 6% in blood.

### Assigning Pathogenicity

The pathogenicity classification of these nine novel variants was determined using the mt-tRNA point mutation pathogenicity scoring system [Yarham et al., 2011], which outlines a number of weighted criteria. There have been no previous reports of the nine variants, all are heteroplasmic, and all are absent from haplogroup-matched controls. Sequential COX–SDH histochemistry revealed COX-deficient fibers in skeletal muscle biopsies from all patients (Fig. 1) with the exception of two—patient 1 (m.4289T>C) and patient 6 (m.8304G>A) where appropriate tissue was unavailable, however for patient 6 numerous COX-deficient skeletal muscle fibers had been previously identified using an individual COX histochemical reaction.

Evolutionary conservation of the affected positions was assessed using the consensus panel of organisms [Yarham et al., 2012]; m.5541C>T, m.5690A>G, m.7451A>T, m.8304G>A and m.16023G>A all affect highly conserved Watson–Crick pairs within stem structures; m.12317T>C affects a highly conserved base in the T loop; m.12206C>T affects the highly conserved discriminator base; m.4289T>C affects a moderately conserved base in the anticodon stem and m.7554G>A affects a less conserved base within the anticodon stem (Supp. Figs. S1 and S2).

Importantly, single-fiber analysis in all patients, with the exception of patients 1 and 6 for whom orientated muscle blocks were not available, demonstrated a statistically significant segregation of mutation load with biochemical deficiency (Fig. 3). In addition, high-resolution northern analysis showed that mt-tRNA^His^ steady-state levels are reduced in skeletal muscle from patient 7 compared with controls (Supp. Fig. S3). On the basis of this evidence, m.5541C>T (11 points), m.5690A>G (11 points), m.7451A>T (11 points), m.12206C>T (16 points), m.12317T>C (11 points), and m.16023G>A (11 points) can all be considered “definitely pathogenic” mutations and are responsible for the clinical presentations observed in these patients.

Despite evidence supporting pathogenicity from single-fiber investigations, the lack of both evolutionary conservation and supportive evidence from biochemical analyses of respiratory chain complex activities means that the m.7554G>A (9 points) cannot currently be considered “definitely pathogenic,” but is classified as “possibly pathogenic.” However, a future report of this variant in a separate family would be sufficient for reclassification as “definitely pathogenic.”

Neither m.4289T>C (7 points) nor m.8304G>A (8 points) have evidence from “gold-standard” functional investigations, and both must consequently be classified as “possibly pathogenic.” Further investigation is therefore required in order to confirm the pathogenic nature of these two variants. Despite the obvious benefits of reporting “definitely pathogenic” mutations, other identified variants that currently lack sufficient evidence should also be reported, along with the appropriate classification as this will enable future reports with additional studies to confirm pathogenicity.

### Molecular Impact

Both m.4289T>C and m.5541C>T occur in genes known to be susceptible to pathogenic mutations (Supp. Table S2), and are located at the Watson-Crick pair adjacent to the anticodon loop (Fig. 2). This position has previously been associated with disease in other mt-tRNAs (m.1630A>G in mt-tRNA^Val^ [Glatz et al., 2011; Horvath et al., 2009a] and m.5628T>C in mt-tRNA^Ala^ [Spagnolo et al., 2001]), and it seems likely that such a mutation might disrupt the secondary structure of the anticodon arm, potentially therefore impacting upon the codon–anticodon interaction. The m.5690A>G (mt-tRNA^Asn^) and m.7554G>A (mt-tRNA^Asp^) variants also occur within the anticodon stem, but in these instances, at the penultimate Watson–Crick pair before the anticodon loop (Fig. 2). Again, this base-pair has been shown to contain pathogenic mutations in other mt-tRNAs including mt-tRNA^Phe^ (m.617G>A [Iizuka et al., 2009]) and mt-tRNA^Ile^ (m.4298G>A [Crimi et al., 2004; Taylor et al., 1998]), indicating the impact of secondary structure disruption on pathogenesis. However, although a number of definitely pathogenic mutations have been found in mt-tRNA^Asn^, none, including the mutation reported here, have yet been confirmed in mt-tRNA^Asp^ (Supp. Table S2).

The m.8304G>A variant in a commonly mutated mt-tRNA gene, *MT-TK* (Supp. Table S2), is located in the D stem (Fig. 2), at a position involved in tertiary structure formation [Helm et al., 2000]. Previously identified pathogenic mutations at this position include m.5521G>A (mt-tRNA^Trp^ [Silvestri et al., 1998]) and m.12147G>A (mt-tRNA^His^ [Melone et al., 2004; Taylor et al., 2004]), confirming the vulnerability of this position. Interestingly, the proband in this case presented with epilepsy, similar to patients with the m.8344A>G *MT-TK* mutation associated with the MERRF phenotype.

The mutation m.16023G>A occurs at position 1 in mt-tRNA^Pro^ (Fig. 2), and although to date, no other definitely pathogenic mutations have been identified at this position, m.8363G>A (mt-tRNA^Lys^) does occur at its cognate pair [Ozawa et al., 1997; Santorelli et al., 1996]. Given the location of this mutation at the end of the acceptor stem, pathogenesis may result from disruption of either posttranscriptional processing of the mt-tRNA from the transcript, or aminoacylation.

The m.7541T>A (mt-tRNA^Ser(UCN)^) mutation is located in the middle of the acceptor stem (Fig. 2) at a position where pathogenic mutations have been identified in other mt-tRNAs including m.642T>C in mt-tRNA^Phe^ [Valente et al., 2009] and m.12201T>C in mt-tRNA^His^ [Yan et al., 2011]). The conserved nature of the pairing at this position suggests that may be important for the maintenance of mt-tRNA secondary structure, and potentially for the binding of aminoacyl-tRNA synthases.

The m.12206C>T mutation occurs at the discriminator base (Fig. 2), and is therefore likely to have a direct impact upon processing of the mtDNA transcript during translation, nucleotidyltransferase activity and aminoacylation. Interestingly, the only other confirmed pathogenic mutation at this position (m.14674T>C in mt-tRNA^Glu^ [Horvath et al., 2009b; Uusimaa et al., 2011]) was found to have lower tRNA steady-state levels rather than excess unprocessed intermediates. This might be explained by a loss of stability conferred by aminoacylation, although a recent study of mitochondrial methionyl-tRNA formyltransferase (*MTFMT*) activity suggests that mt-tRNAs may actually primarily exist in the uncharged form, at least in the case of mt-tRNA^Met^ [Tucker et al., 2011].

Finally, the m.12317T>C mutation occurs in the T loop, adjacent to the T stem (Fig. 2). This region of mt-tRNAs is highly variable, yet the high evolutionary conservation of this position (Supp. Fig. S2) implies an important role for this particular base. Given that this position in mt-tRNA^Leu(CUN)^ is involved in tertiary interactions that confer a structurally important turn in the phosphodiester backbone, a substitution at this position may impact upon the essential shape of the anticodon loop.

The molecular mechanisms by which the mutations described here actually cause disease are only speculation at this stage, based upon the location of the changes and observations made about similarly positioned mutations in other mt-tRNAs. Given the identification of mutations in genes encoding proteins involved in mt-tRNA modification as a cause of mitochondrial disease (e.g., *PUS1* [Bykhovskaya et al., 2004], *TRMU* [Zeharia et al., 2009], *MTO1* [Ghezzi et al., 2012], *MTFMT* [Tucker et al., 2011], the disruption of these post-transcriptional modifications represents a major consideration for potential disease mechanisms. Modifications such as pseudouridine and methyl groups are highly prevalent in tRNA species and are fundamental to structure and function—for instance modification of position 34 (the wobble position) is crucial to determining mt-tRNA specificity [Suzuki et al., 2011]. Of the mutations reported here, only m.8304G>A is known to be located at a position normally modified (m^2^G10), although some of the others occur at residues known to be modified in different mt-tRNAs (not all species have yet been characterized). The impact of a loss of this particular modification is currently unclear but structural disruption seems plausible. Further functional studies are therefore essential to fully elucidate the mechanisms by which these mutations result in disease.

### Clinical and Diagnostic Implications

Point mutations within mt-tRNA genes represent the most common group of mtDNA mutations to cause human disease, yet many questions remain with regards understanding the critical factors which underlie their clinical expression, inheritance, and even the permissive susceptibility of these genes to mutation. We present data on nine novel heteroplasmic mt-tRNA variants, each resulting in a different clinical phenotype, highlighting the lack of a firm genotype–phenotype correlation in mt-tRNA disease. Furthermore, each novel mutation affects a different mt-tRNA molecule, although an assessment of all well-characterized mt-tRNA mutations associated with disease reveals that some mt-tRNAs—in particular mt-tRNA^Leu(UUR)^, mt-tRNA^Ile^, and mt-tRNA^Lys^—harbor more mutations than others (Supp. Fig. S2).

Other questions persist; recent data have suggested that those mt-tRNA mutations which exert a major biochemical phenotype in dividing cells are unlikely to be inherited and transmitted through the female germline [Elson et al., 2009], yet what are the factors that determine how these mutations arise sporadically? The importance of sequencing mtDNA from a clinically relevant tissue and extensive screening of maternally related individuals to investigate mutation inheritance and segregation are highlighted within this article. Furthermore, although there are some loose correlations with clinical phenotype and mutation of a specific mt-tRNA gene (e.g., mt-tRNA^Ile^ mutations and cardiomyopathy, mt-tRNA^Lys^ mutations and the MERRF phenotype), the data presented here reminds us how phenotypically diverse mt-tRNA mutations are and that only a small number (as exemplified by the m.3243A>G and m.8344A>G mutations) account for a substantial proportion of all mtDNA disease diagnoses, whereas others are limited to singleton families.

**Figure 4:**
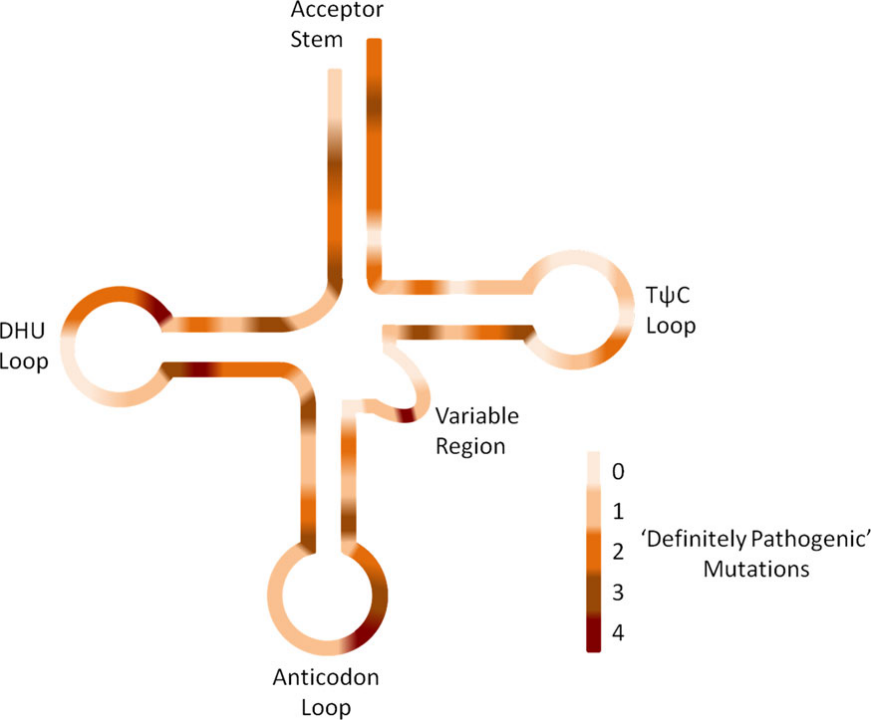
Location of “definitely pathogenic” mutations across the mt-tRNAs. The number of “definitely pathogenic” mt-tRNA point mutations found at various locations throughout the cloverleaf are shown by the use of a gradient color scale; lighter colors equate to fewer mutations, whereas darker colors specify more mutations. “Definitely pathogenic” mutations occur mainly in the stems and to a lesser degree in the loops with the notable exception of a position in the DHU-loop, which includes the m.3243A>G *MT-TL1* mutation, and the third base of the anticodon.

What makes a particular site or nucleotide within an mt-tRNA molecule vulnerable to mutation? Based on our reanalysis of the pathogenicity of reported human mt-tRNA mutations [Yarham et al., 2011], we have updated the analysis of McFarland and colleagues who reported mt-tRNA mutation “hotspots” in both the anticodon and acceptor stems, on the basis that 73% of all reported mt-tRNA mutations occurred in stem structures, with 94% of these disrupting Watson–Crick base pairing [McFarland et al., 2004]. The current dataset confirms that these trends hold true, with 67% of the 116 reported mt-tRNA mutations which have been reclassified as “definitely pathogenic” occurring with the stem structures and of these, 90% result in the disruption of Watson–Crick base pairing (Fig. 4).

## Conclusion

Over 200 mitochondrial tRNA variants have now been reported. The fact that only half of these variants have sufficient evidence to be “definitely pathogenic,” demonstrates the difficulties faced when characterizing potentially pathogenic variants. The vast heterogeneity in the phenotype–genotype relationship is demonstrated by the nine patients reported here, making accurate diagnosis immensely difficult and reaffirming the importance of meticulous clinical assessment, appropriate tissue selection for analysis, and comprehensive laboratory investigations. The variants reported here should be added to the library of mt-tRNA variants associated with disease, contributing to our knowledge of this field and confirming mt-tRNA mutations as an important subgroup of human genetic disorders [Schon et al., 2012].
