# Supplementary material for: Pathogenic Mitochondrial tRNA Point Mutations: Nine Novel Mutations Affirm Their Importance as a Cause of Mitochondrial Disease
Source: Hum Mutat. 2013 Aug 14;34(9):1260–8. doi: 10.1002/humu.22358 (PMC3884772; doi:10.1002/humu.22358)
Supplement: Supplementary file 1 [file humu0034-1260-sd1.pdf]

<u>Supp. Document S1. Patient clinical summaries</u>

Patient 1 (m.4289T>C *MT-TI* mutation)

This 47 year-old lady had a normal early childhood, but developed diabetes mellitus at age 8 years. At age 11 years routine diabetic eye surveillance revealed retinitis pigmentosa (RP). By her early twenties ataxia was a significant problem, impairing her mobility. The ataxia and RP progressed so that by her late twenties she had no functional vision and was wheelchair dependent. A progressive cognitive impairment has been noted over the last 15 years and she has increasing difficulty with speech and swallowing. Her mother and two sisters do not share any similar clinical problems.

Patient 2 (m.5541C>T *MT-TW* mutation)

This 39 year-old gentleman first presented at age 30 years with a stroke-like episode involving the right occipital lobe. Further stroke-like episodes have been associated with focal status epilepticus with involvement of the right temporal and both occipital lobes. Infarction of the occipital lobes has resulted in cortical visual impairment. There is a maternal family history of hypertension but no history of stroke, deafness or diabetes in the extended family.

Patient 3 (m.5690A>G *MT-TN* mutation)

This 45 year-old lady first noted an asymmetric (right sided) ptosis at age 14 years. This progressed rapidly, involving the left eye and requiring bilateral surgery by age 20 years. Over the same period she also suffered from frequent, monthly, migraines, which persist, but now occur much less often. At the age of 39 years she experienced a transient weakness and paraesthesia involving the entire right side of her body, which evolved over a period of 5 days and slowly resolved. There were no associated cranial MRI abnormalities. Fatigue and generalized weakness are prominent features of her illness. Family history is unremarkable save for her mother, who has recently developed ptosis and dementia. Two daughters are healthy with no ptosis. Examination reveals bilateral ptosis, worse on the right, and chronic progressive external ophthalmoplegia. There was evidence of both proximal and distal myopathy with proximal being more severe.

Patient 4 (m.7451A>T *MT-TS1* mutation)

This 29 year old gentleman has had progressive bilateral ptosis since the age of 15 years and undergone corrective surgery. Eye movements are moderately impaired in all directions and he suffers from diplopia on left and right lateral gaze. He has no evidence of proximal myopathy and continues to engage in regular aerobic exercise, recently completing a marathon in 3hours and 38 minutes. He has a concurrent diagnosis of Crohn's disease.

Patient 5 (m.7554G>A *MT-TD* mutation)

This 34 year-old right handed gentleman first developed problems of clumsiness and unsteadiness, with frequent falls, in early childhood. He was noted to have pes cavus but no evidence of peripheral neuropathy. A melanoma was removed from his left leg at age 13 years, but his history was otherwise unremarkable. On examination he had a broad based gait with valgus deformity of the knees. He had broken visual pursuit but vision and eye examination were otherwise normal. There was evidence of proximal muscle weakness but no contractures. Creatine kinase was mildly elevated at 544 IU. Regarding family history, his father had a kyphosis, his mother was of short stature and his sister had a diagnosis of Multiple Sclerosis.

Patient 6 (m.8304G>A *MT-TK* mutation)

This gentleman first presented at age 7 years with hearing loss that progressed rapidly so that bilateral hearing aids were necessary by age 9 years and he has subsequently had a cochlear implant at age 21 years. He has always been considered thin, but following the development of dysphagia, in his twenties, a gastrostomy was inserted to facilitate adequate nutrition. At age 12 years he had his first seizure. A subsequent EEG was abnormal and he was commenced on sodium valproate. His vision has been impaired by a retinal bleed at age 21 years and the later development of cataracts, though these have now been excised. Examination reveals head titubation that is not associated with limb tremor or ataxia, but he does have nystagmus and complains of oscillopsia. In addition to these problems he also has a history of osteogenesis imperfecta and hypogonadotrophic hypogonadism.

Patient 7 (m.12206C>T *MT-TH* mutation)

This 52 year-old gentleman first presented at age 42 years when he was admitted to hospital with focal seizures associated with visual aura and secondary generalization. He was noted to have a

disinhibited affect, significant cognitive impairment and a right hemianopia. Two weeks after admission he developed a sensory impairment in his left arm and lost dexterity of finger movement in that hand. Investigations revealed a mild hyponatraemia that became worse following the introduction of Carbamazepine; subsequently withdrawn. Visual evoked responses were delayed. Serial cranial MRI scans revealed infarcts in the right occipital and right temporal lobes with the latter subsequently extending to the Sylvian fissure. A third area of infarction occurred in the right posteromedial thalamic area; this correlated in time and anatomical location with the onset of symptoms in his left arm. His clinical course has been one of progressive decline with recurrence of seizures and a deterioration in cognitive function.

There is a positive family history in that a 45 year-old brother presented at the age of 21 years with headache, seizures and a right temporoparietal infarct. Bilateral optic atrophy and long tract signs were noted on examination. Seizures proved difficult to control and as with his brother he developed progressive cognitive decline. A sister died at the age of 41 years following a sudden illness necessitating liver transplantation.

Patient 8 (m.12317T>C *MT-TL2* mutation)

This gentleman had a history of bilateral ptosis from late childhood with subsequent chronic progressive external ophthalmoparesis evident from early adult life. He developed non-insulin dependent diabetes in his thirties before presenting at the age of 48 years with a slowly progressive proximal myopathy and associated fatigue. Following a suspected myocardial infarction in his sixties, cardiac failure with predominantly left ventricular dysfunction was diagnosed. Despite initial improvement in cardiac status following treatment, he died in his early seventies.

Patient 9 (m.16023G>A *MT-TP* mutation)

This lady presented at age 35 years with migraine headache and a history of bilateral sensorineural deafness from her twenties. Examination revealed deafness and pigmentary retinopathy, but no other positive findings. A cranial MRI scan showed multiple areas of white matter high signal intensity on T2 imaging. There has been little evidence of disease progression on follow-up. There is a strong family history of both migraine and deafness.

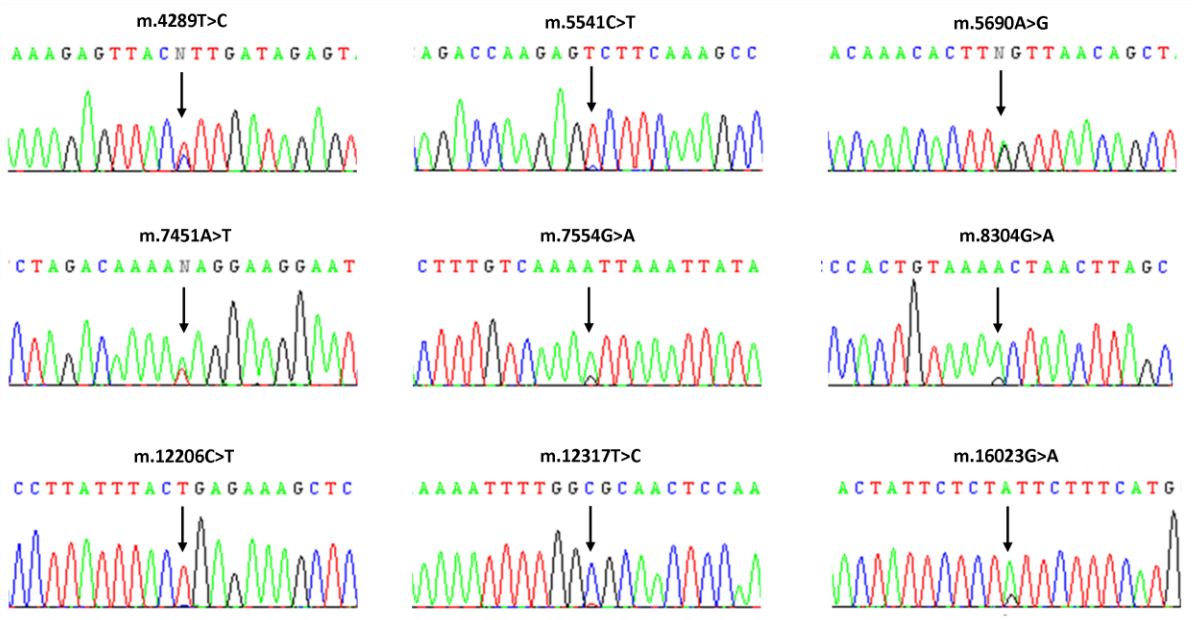

Supp. Figure S1. Sequencing Chromatograms. The sequencing chromatograms from each of the 9 patients reveal the mt-tRNA point mutations found in each (labelled with an arrow).

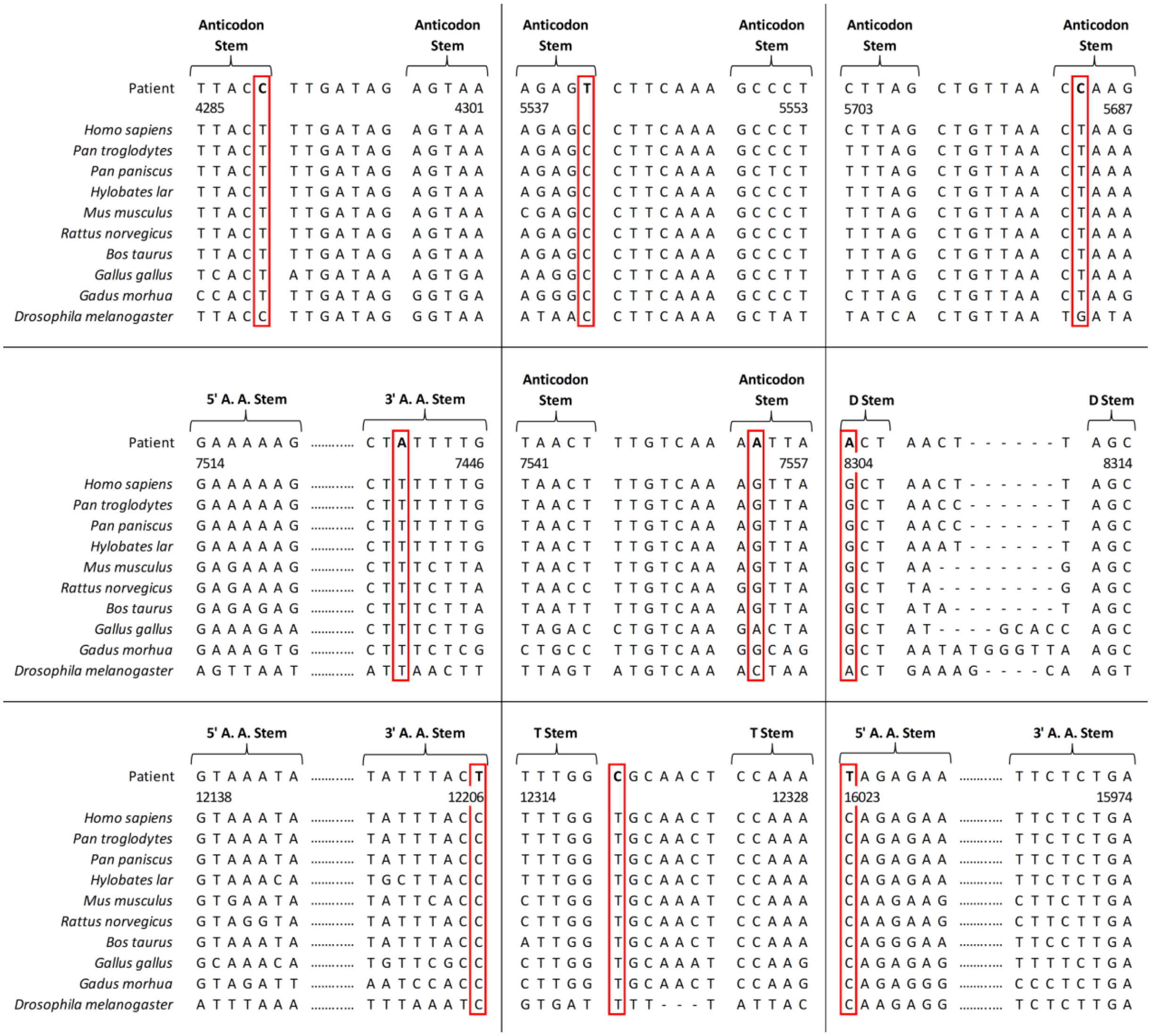

Supp. Figure S2. Evolutionary conservation. Evolutionary conservation panels appropriate for each mutation are shown, with the affected position highlighted by the red box across the species.

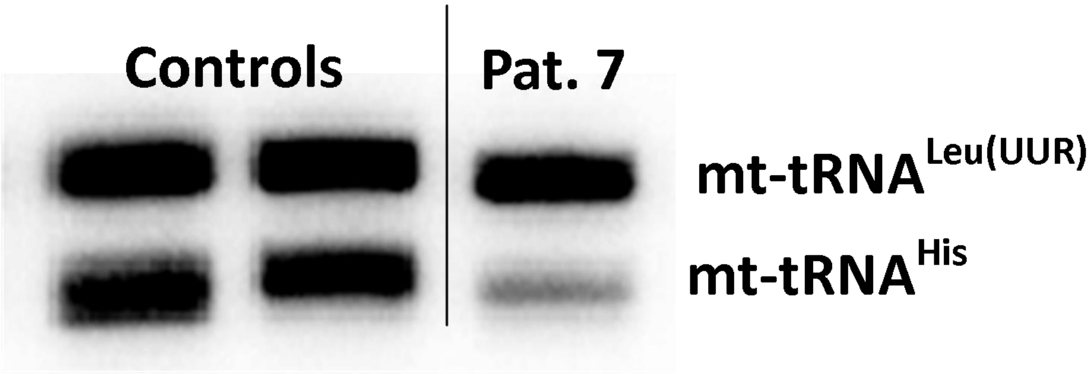

Supp. Figure S3. Quantification of mt-tRNA steady-state levels by high resolution northern blot analysis. Assessment of mt-tRNA^{His} steady-state levels in skeletal muscle from patient 7 is shown, demonstrating ~25% of control mt-tRNA^{His} steady-state levels.

Supp. Table S1. Pyrosequencing of Novel Variants

Mutation	Gene Name (ID)	Forward Amplification PCR Primer		Reverse Amplification PCR Primer		Pyrosequencing Primer	
		5' Position	Sequence	5' Position	Sequence	5' Position	Sequence
4289T>C	*MTTI* (4565)	4244	5' GCATTCCCCCTCAAACCTAA 3'	4334	Bio-5' TCCTAGAAATAAGGGGGTTTAAGC 3'	4269	5' ATGTCTGATAAAAGAGTTAC 3'
5541C>T	*MTTW* (4578)	5368	Bio-5' CCTCAATCACACTACTCCCCATAT	5607	5' AGTGGGGTTTTGCAGTCCTTAG 3'	5559	5' TTACTGAGGGCTTTGAA 3'
5690A>G	*MTTN* (4570)	5650	Bio-5' GCCCTTACTAGACCAATGGGACT 3'	5739	5' GCGGGAGAAGTAGATTGAAGC 3'	5709	5' AGGGTGCTTAGCTGTTA 3'
7451A>T	*MTTS1* (4574)	7411	Bio-5' ACACATTCGAAGAACCCGTATACA 3'	7600	5' CGCTGCATGTGCCATTAAGATATA 3'	7468	5' GGGTTCGATTCCTTC 3'
7554G>A	*MTTD* (4555)	7526	5' AGAAAAACCATTTCATAACTTTGTC 3'	7599	Bio-5' GCTGCATGTGCCATTAAGATATA 3'	7534	5' CATTTCATAACTTTGTCAA 3'
8304G>A	*MTTK* (4566)	8250	5' GGCCCGTATTTACCCTATAGCAC 3'	8371	Bio-5' GGGCATTTCACTGTAAAGAGGTGT 3'	8286	5' TCTAGAGCCCACTGTAAA 3'
12206C>T	*MTTH* (4564)	12118	Bio-5' CATTACCGGGTTTTCCTCTTG 3'	12220	5' GGGGCATGAGTTAGCAGTTCT 3'	12209	5' CAGTTCTTGTGAGCTTTC 3'
12317T>C	*MTTL2* (4568)	12293	Bio-5' GGTCTTAGGCCCCAAAAATT 3'	12450	5' GGATTTTACATAATGGGGGTATGA 3'	12340	5' TTATTACTTTTATTTGGAGTTG 3'
16023G>A	*MTTP* (4571)	15986	Bio-5' GCACCCAAAGCTAAGATTCTAATT 3'	16083	5' GGTTGTTGATGGGTGAGTCAAT 3'	16041	5' TGCTTCCCCATGAAA 3'

This table displays details of the primer sequences used for mutation load analysis by pyrosequencing. Each of the mutations is described along with the Gene Name and ID. Gene sequences were determined and numbered according to the revised Cambridge Reference Sequence (rCRS), NC_012920.1. Forward and reverse primer pairs were designed for mtDNA amplification prior to pyrosequencing analysis. One primer in each pyrosequencing primer pair had a 5' biotinylation modification (Bio-5'). An additional, sequencing primer was required for pyrosequencing analysis, which was used to assess mutation load.

Supp. Table S2. Distribution of reported mutations in all mt-tRNAs

mt-tRNA	Definitely Pathogenic Mutations	All Reported Mutations
Phe	8	17
Val	4	11
Leu(UUR)	15	29
Ile	13	22
Gln	2	6
Met	3	5
Trp	9	15
Ala	4	6
Asn	7	8
Cys	1	6
Tyr	5	6
Ser(UCN)	8	14
Asp	0	4
Lys	10	22
Gly	1	5
Arg	2	5
His	5	6
Ser(AGY)	4	8
Leu(CUN)	9	15
Glu	9	13
Thr	0	10
Pro	3	7
**Total:**	**122**	**240**

The distribution of definitely pathogenic mutations (according to the scoring system, Yarham *et al.* 2011) and all reported mutations in the literature across the 22 mt-tRNAs is shown. This data include the 9 mutations reported in this publication and highlights the continued over-representation of mutations in mt-tRNA$^{Leu(UUR)}$, mt-tRNA^{Ile} and mt-tRNA^{Lys}.
